# Genetic characterisation of variants of the virulence plasmid, pSLT, in *Salmonella enterica* serovar Typhimurium provides evidence of a variety of evolutionary directions consistent with vertical rather than horizontal transmission

**DOI:** 10.1371/journal.pone.0215207

**Published:** 2019-04-11

**Authors:** Lester Hiley, Rikki M. A. Graham, Amy V. Jennison

**Affiliations:** Public Health Microbiology, Forensic and Scientific Services, Queensland Department of Health, Coopers Plains, Queensland, Australia; Institut National de la Recherche Agronomique, FRANCE

## Abstract

The virulence plasmid pSLT as exemplified by the 94 Kb plasmid in *Salmonella* Typhimurium strain LT2 is only found in isolates of serovar Typhimurium. While it occurs commonly among such isolates recent genotyping methods have shown that it is mostly confined to certain genotypes. Although pSLT plasmids are capable of self-transmissibility under experimental conditions their confinement to certain host genotypes suggests that in practice they are maintained by vertical rather than by horizontal transmission. This would imply that evolution of the pSLT plasmid proceeds in parallel with evolution of its host. The development of a phylogenetic evolutionary framework for genotypes of *S*. Typhimurium based on single-nucleotide-polymorphism (SNPs) typing provided an opportunity to test whether the pSLT plasmid coevolves with its host genotype. Accordingly SNPs analysis was applied to the pSLT plasmids from 71 strains *S*. Typhimurium of Australian and international origins representing most of the genotypes which commonly have a pSLT. The phylogenetic tree showed that pSLT sequences clustered into almost the same groups as the host chromosomes so that each pSLT genotype was associated with a single host genotype. A search for tandem repeats in pSLT plasmids showed that a 9 bp VNTR in the *traD* gene occurred in the pSLT from all isolates belonging to Clade II but not from isolates belonging to Clade I. Another 9 bp repeat occurred only in three Clade I genotypes with a recent common ancestor. The evidence relating to both of these VNTRs supports the proposition that the pSLT plasmid is only transmitted vertically. Some isolates belonging to one *S*. Typhimurium genotype were found to have pSLTs which have lost a large block of genes when a resistance gene cassette has been acquired. Examples were found of pSLT plasmids which have recombined with other plasmids to form fusion plasmids sometimes with loss of some pSLT genes. In all cases the underlying genotype of the modified pSLT was the same as the genotype of regular pSLTs with the same host genotype implying that these changes have occurred within the host cell of the pSLT plasmid.

## Introduction

The virulence plasmid of *Salmonella enterica* serovar Typhimurium (*S*. Typhimurium), designated pSLT [1], is, in its most common form, a large, 94Kb, low-copy-number plasmid which belongs to the IncFII_s_ plasmid incompatibility group [2] and occurs commonly amongst isolates of *S*. Typhimurium [3]. The plasmid has an 8-Kb region which encodes the *spv* genes thought to give a competitive advantage to the host by increasing the growth rate of the bacterium during the systemic phase of disease [4]. In its most common form it does not have any antimicrobial resistance genes but forms of pSLT with resistance genes such as pSal8934B (GenBank Acc. No. NC_019109), pSTU288-1 (NC_021155) and pSTMDT12_L (NC_016861) have been recorded.

The pSLT plasmid was first reported in 1970 [5] but it was not shown to be self-transmissible until 1999 [6]. Under optimal experimental conditions the pSLT plasmids of *S*. Typhimurium strains LT2 and 14028 were self-transmissible at a rate of 2.9 x 10^−4^ transconjugants/donor [6]. This was in the range reported for most natural self-transmissible plasmids of between 10^−3^ and 10^−5^ [7]. A much lower rate of self-transmissibility (10^−9^) was found for strain SL1344 pSLT [8]. In the case of SL1344 pSLT (NC_017720) it was shown that the low rate of transmission is due to a single C to T nucleotide mutation in the *traG* gene causing an A to V amino acid substitution in the TraG protein [8]. Conjugal transfer of pSLT from *S*. Typhimurium strain ATCC 14028 to a Typhimurium strain lacking pSLT under natural conditions in the distal portion of the mouse small intestine has been demonstrated [9]. Despite this evidence the very limited host range of pSLT implies that conjugal transfer of pSLT is rare in practice. pSLT is not found in genera other than *Salmonella* and it is only found in serovar Typhimurium. Related virulence plasmids are found in eight *Salmonella* serovars including Paratyphi C, Enteritidis, Dublin, Gallinarum/Pullorum and Choleraesuis [10]. All of these plasmids lack some of the genes of the *tra* operon and are consequently non-conjugative. They are therefore likely to be maintained by vertical rather than horizontal transmission. This was confirmed when it was shown that, with the exception of serovar Enteritidis, the virulence plasmids from the named serovars as well as from serovar Typhimurium have the same phylogenetic relationships as the host chromosomes. Plasmid pSENV from serovar Entertidis was shown to have been inherited horizontally because it was more similar to plasmids from more distantly related serovars Typhimurium, Choleraesuis and Paratyphi C than to those from the very closely related serovars Dublin and Gallinarum/Pullorum [10].

Genotyping of *S*. Typhimurium isolates by a combination of variable-number tandem repeats (VNTRs) and clustered regularly interspaced short palindromic repeats (CRISPR) profiling called Repeats Typing has been used by Hiley et al 2014 [11] to recognise at least fifteen distinct genotypes. Whole genome sequencing (WGS) followed by core-genome single-nucleotide-polymorphism (SNP) analysis has supported the separation of strains into the same genotypes and determined the likely relationships between genotypes [12]. Genotyping has shown that there is a distinct divide between genotypes likely to have pSLT and those unlikely to have it. Isolates belonging to ten genotypes nearly always have a pSLT indicated by the presence of a marker located on the pSLT, called STTR10pl, used in multi-locus-VNTR-analysis (MLVA) typing of *S*. Typhimurium isolates [13] while isolates belonging to five genotypes nearly always lack STTR10pl and presumably have no pSLT [11]. This strong association between genotype and possession or otherwise of pSLT plasmid infers that maintenance of pSLT in *S*. Typhimurium is almost entirely dependent on vertical transmission rather than horizontal transmission. If the pSLT plasmid is almost totally confined to its host then it should be possible to show that its evolution follows a similar course to that of its host. In time the evolved forms of pSLT should become specific to the evolved genotype of its host. We have therefore applied SNPs analysis to investigate the phylogeny of pSLT plasmids within various genotypes of *S*. Typhimurium to determine whether pSLT co-evolves with its host or whether sequence variants of pSLT are randomly distributed across genotypes.

In addition to single-nucleotide mutations, evolution of a plasmid can occur as large-scale changes such as insertions, deletions, inversions and translocations often driven by mobile elements such as transposons and IS-elements [14]. VNTRs can also arise in plasmids and may provide evidence of on-going evolution. We therefore compared repeat numbers for the genotypes which have a 9 bp VNTR located in the *traD* gene (PSLT104 in LT2 pSLT). Testing for VNTRs in pSLT allowed us to discover a new example of pSLT evolution caused by an insertion sequence (IS). We found that some isolates belonging to one genotype lacked the STTR10pl VNTR but they nevertheless had the 9 bp VNTR in the *traD* gene of pSLT. They were found to have a modified pSLT in which loss of genes (including the STTR10pl VNTR site), resulting from the acquisition of antimicrobial resistance genes involving IS*26*, has occurred. Another way plasmids have been shown to evolve is by recombination of plasmids to produce fusion plasmids [15]. We report examples of pSLT in fusion with plasmids belonging to other incompatibility groups.

## Results and discussion

### SNPs analysis of pSLT plasmids in different host genotypes

**Fig 1:**
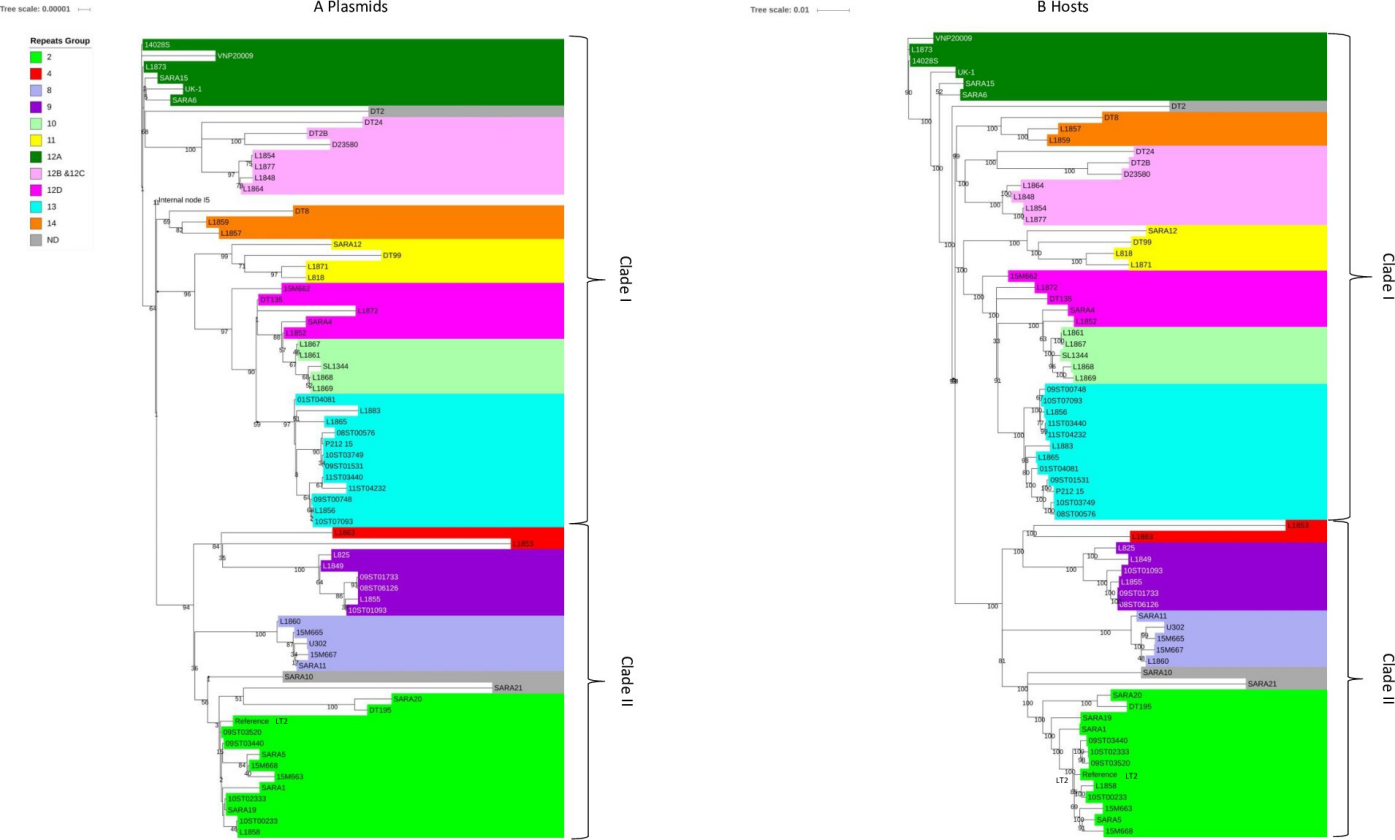
**Maximum-likelihood phylogeny based on full length sequence SNPs analysis for 71 pSLT plasmids from Australian and international isolates of *S*. Typhimurium (A) compared with that for the host chromosomes based on core SNPs analysis (B).** The scale bar shows the proportion of total substitutions for the site. The legend indicates the lineages (Repeats Groups) for the clusters according to Fu et al, 2017 [12].

Isolates were chosen to represent almost all of the genotypes which commonly have a pSLT plasmid. We chose 50 international and Australian isolates from the panel recruited for the comparative genomics of *S*. Typhimurium study of Fu et al 2017 [12] (S1 Table and S3 Table) as well as another 21 international and Australian isolates with pSLT plasmids and known genotype which have been sequenced as part of this study (S2 Table). All these isolates belonged to Clades I and II because pSLT is almost always absent from Repeats Group 3 (RG3), RG5 and RG6 genotypes [11] [11, S1 and S2 Figs] in Clades III, IV and V [12]. The pSLT phylogenetic tree showed clusters of pSLT sequences (Fig 1A) almost identical to the clusters of the corresponding Typhimurium host isolates (Fig 1B). There were no examples of pSLT plasmids from one host genotype locating close to plasmids which came from host genotypes more distantly related as happens with the horizontally acquired virulence plasmid pSENV in *S*. Enteritidis [10]. The inferred phylogenetic relationships between pSLT clusters were very similar to those for the host isolate clusters. For instance, the pSLTs and the host genomes for SARA4 and L1852 locate next to the clusters for RG12D and RG10. The host CRISPR profiles for these two isolates are a hybrid combination of RG12D CRISPR 1 and RG10 CRISPR 2 [12]. In another example, one of the three pSLT SNPs common only to isolates belonging to RG10, RG12D and RG13 clusters was the SNP identified in the *traG* gene of strain SL1344 (RG10) which greatly reduces the transmissibility of pSLT [8]. With the exception of 15M662 belonging to RG12D all the pSLT sequences in these three clusters also had a 9 bp repeat present in SL1344 as reported below.

This result gives support to the proposition that the pSLT plasmid is not transmitted between genotypes of *S*. Typhimurium and that evolution of the plasmid occurs in parallel with evolution of the host so that all the pSLT sequences making up a pSLT genotype come from hosts which belong to a single *S*. Typhimurium genotype.

### Possible evolutionary trends indicated by VNTRs in pSLT plasmids

The range of tandem repeat numbers in a VNTR may vary from one genotype to another and may be indicative of evolutionary trends as populations diverge into different ecological niches. We have examined the range of repeat numbers for a pSLT VNTR which occurs in all genotypes in Clade II with a pSLT but not in those in Clade I [12]. This is a 9 bp VNTR located in the *traD* gene, PSLT104 in LT2. Repeat numbers varied from 5 to 19 amongst 220 Queensland isolates genotyped by MLVA profiling. There were some distinct differences in the range of repeat numbers among the different genotypes (Table 1). The highest number was seen in the RG4B genotype. The numbers in RG2 genotypes were usually higher than for RG9A and RG9B genotypes while in DT89 isolates belonging to RG4A genotype numbers were higher than for other members of RG4A. This seems to indicate that, for certain genotypes, there is a relationship between the number of repeats for this VNTR and the host genotype. Isolates in Clade I only ever had a single 9 bp sequence in their pSLT. We infer from this evidence that the pSLT plasmid has not undergone conjugative transfer between genotypes belonging to different clades. It may be that this pSLT VNTR only appeared in Clade II genotypes after Clade I split from a common ancestor for Clades I and II. This is a new observation which is supported by reports of other distinct differences between Clade I and Clade II genotypes in chromosomal VNTRs as well as CRISPR composition [11] [12].

**Table 1:** Number of isolates of *S*. Typhimurium with pSLT plasmids with indicated number of *traD* 9 bp repeats for nominated genotypes of Clade II.

	Number of 9 bp repeats															
Genotype*	5	6	7	8	9	10	11	12	13	14	15	16	17	18	19	Total
**RG2**		1		1	1	4	22	16	7	2						54
**RG4A**		5	1													6
**RG4A/DT89**						14										14
**RG4B**														1	1	2
**RG8**			2	2			2	2	2	4	2		1			17
**RG9A**	3	81	20	13	1	1										119
**RG9B**		2	4	1			1									8

* pSLT plasmids from 105 Clade I isolates representing all Clade I genotypes had 1 repeat only

A duplication of the 9 bp sequence GGCCTGCAT was identified at nt 69568–69588 in the pSLT (NC_017720) from *S*. Typhimurium strain SL1344 belonging to RG10. In LT2 pSLT (AE006471) a singleton 9 bp sequence is located at nt 24257–24265 (in reverse) in the intergenic sequence between PSLT033 and PSLT034. Further investigation showed that this duplicated sequence was present only in all of the available pSLT sequences in strains belonging to RG10 (including two RG12D/RG10 hybrids), RG12D and RG13 except for strain 15M662 in RG12D. These three genotypes have been noted to descend from a recent common ancestor according to the core SNP phylogenetic tree generated by Fu et al 2017 [12].

### VNTRs discover a modified pSLT in RG2 isolates

Absence of STTR10pl product in the MLVA profile for a *S*. Typhimurium isolate is considered a proxy for the absence of the pSLT plasmid. It has been found that all or nearly all isolates belonging to a single genotype either have STTR10pl or it is absent. However for the RG2 genotype (to which LT2 belongs) we found 27 out of 93 isolates belonging to our local collection of DT141 or DT141 var 1 phage types lacked the STTR10pl VNTR. In the course of conducting tests for the 9 bp VNTR in *traD* gene of pSLT we found that eighteen out of twenty DT141/141 var 1 isolates without STTR10pl product had the 9 bp VNTR in the *traD* gene. Furthermore they nearly all exhibited resistance to ampicillin (AMP), streptomycin (STR) and sulphonamide (SUL) whereas most DT141/141 var 1 isolates with STTR10pl showed no antibiotic resistance. It was evident that the resistance profile of the DT141 isolates lacking the STTR10pl VNTR but with the 9 bp VNTR was linked to the VNTR profile (χ^2^ = 66.58, p < .01).

**Fig 2:**
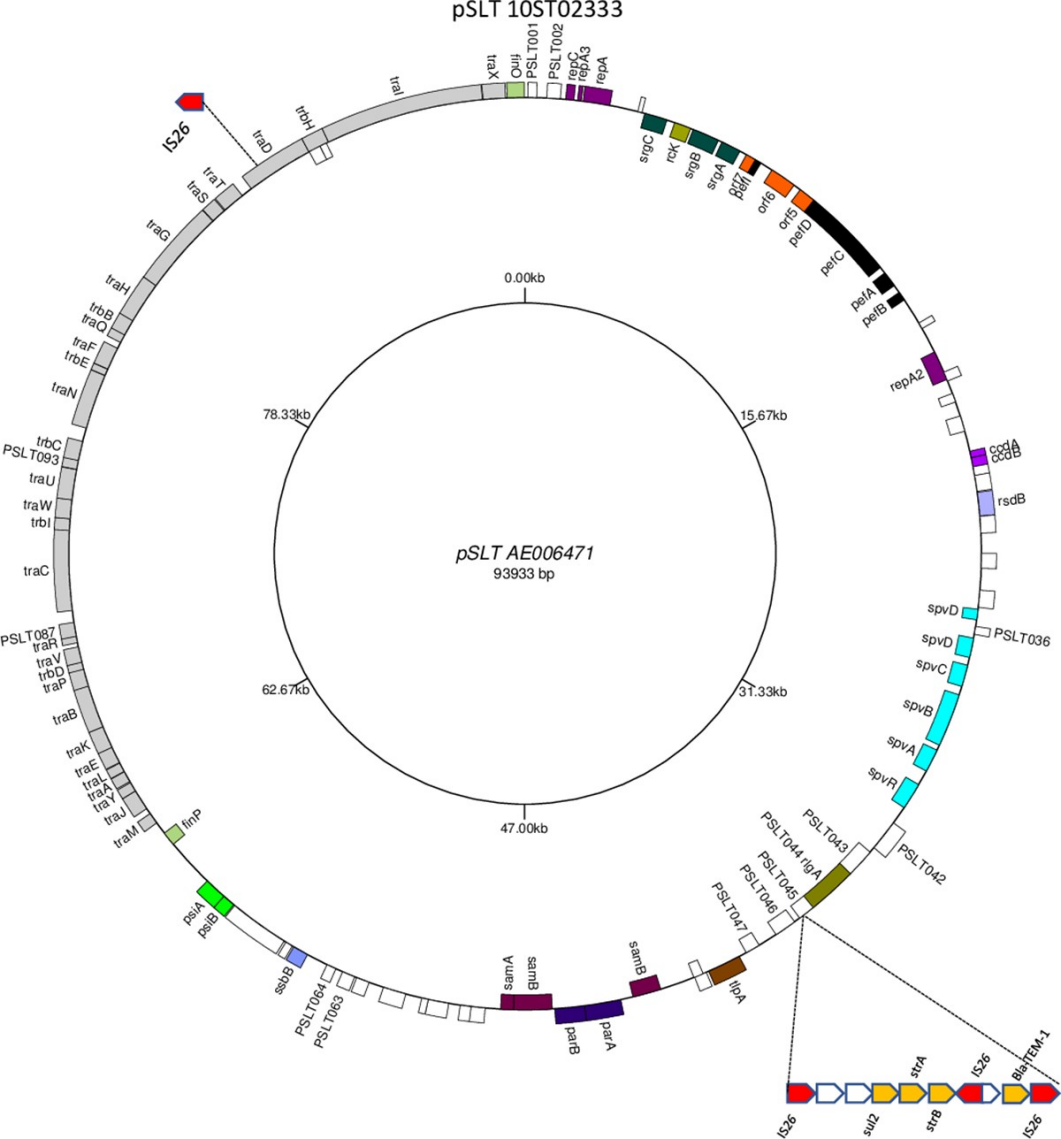
Theoretical structure of 103513 bp pSLT plasmid of 10ST02333 shown as modifications to the gene map sequence of LT2 pSLT AE006471. The antibiotic resistance cassette is shown as an insert in gene PSLT045 and another IS*26* is shown inserted into the *traD* gene. Figure drawn using GenomeVx http://wolfe.ucd.ie/GenomeVx/ [17].

In order to elucidate further the status of the pSLT plasmid in DT141 isolates with the 9 bp VNTR in the *traD* gene but without the STTR10pl VNTR we selected primers to various genes around the LT2 pSLT genome (S4 Table) including genes PSLT064 and 065 flanking the site of the STTR10pl VNTR and genes PSLT103 and 105 flanking the *traD* gene and conducted PCR testing on DT141 isolates with and without the STTR10pl VNTR. Isolates without STTR10pl differed from isolates with STTR10pl by failing to show PCR product for selected genes somewhere after PSLT052 and before PSLT105 indicating deletion of a large number of genes from the pSLT. We conducted WGS on three DT141 isolates, one with STTR10pl and no antibiotic resistance, a rare one with STTR10pl and the AMP STR SUL resistance profile and one with no STTR10pl but with the AMP STR SUL resistance profile. *De novo* assembly of the sequences for the isolate with STTR10pl but no antibiotic resistance (09ST03440, S2 Table) yielded a single contig 93986 bp in size which differed in sequence from LT2 pSLT by one SNP (excluding differences in repeat numbers for the two VNTRs). *De novo* assembly of the sequences for the isolate with STTR10pl and the AMP STR SUL resistance profile (10ST02333, MLVA 5-14-9-10-0211, S2 Table) produced five contigs of interest. One contig, A, spanned LT2 pSLT genes PSLT046 to within PSLT104 (*traD*) at 100% identity. Another contig, B, spanned LT2 pSLT genes from within PSLT104 to PSLT045 with one SNP difference. Another contig, C, contained *sulII*, *strA* and *strB* resistance genes as part of a resistance cassette and another contig, D, contained a *bla*-TEM-1 gene. The fifth contig was the 820 bp IS element, IS*26* [16]. A theoretical construct for the 103513 bp pSLT of 10ST02333 was determined as detailed in S1 Text and as represented in Fig 2.

**Fig 3:**
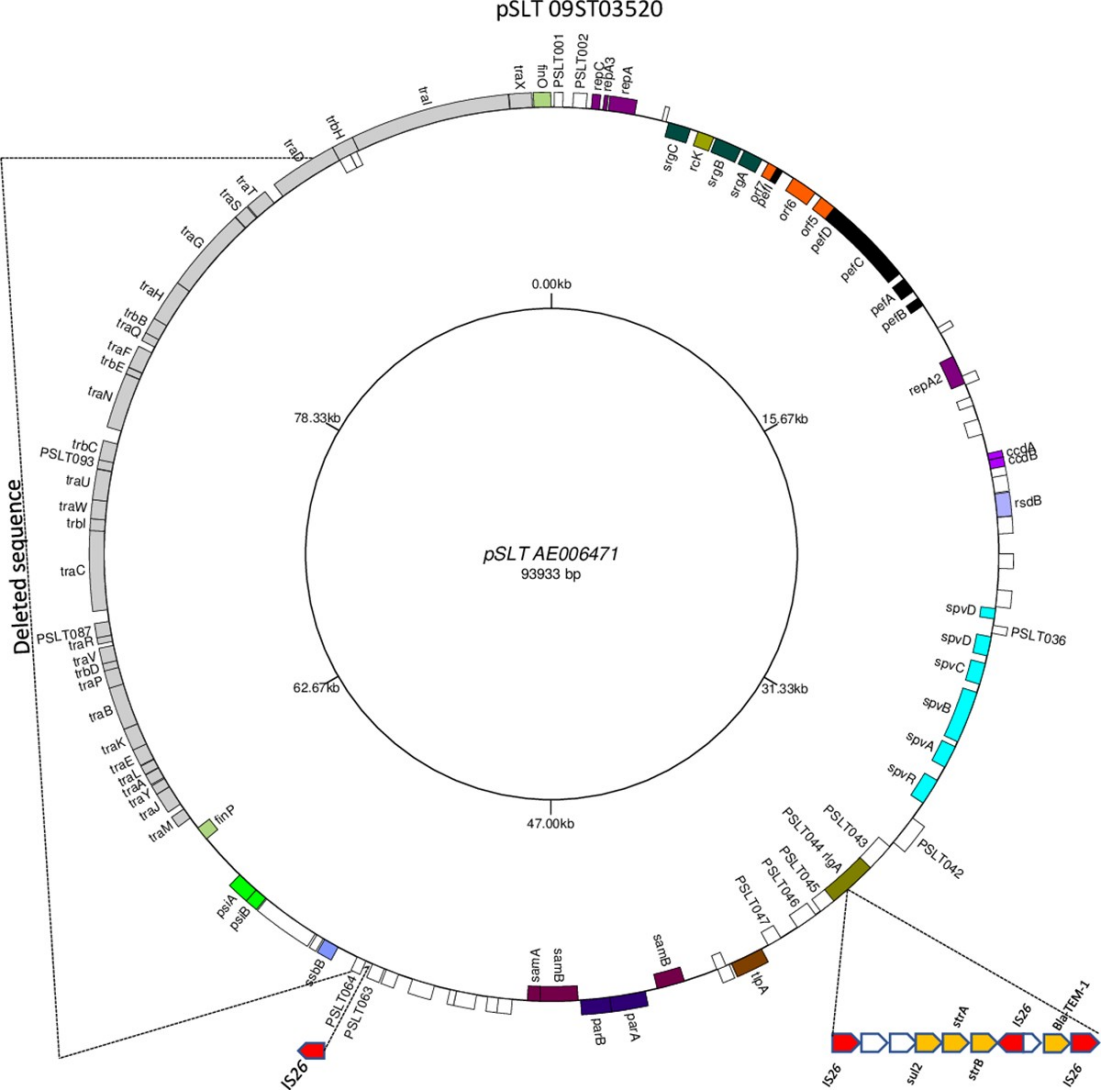
Theoretical structure of 71167 bp pSLT plasmid of 09ST03520 shown as modifications to the gene map sequence of LT2 pSLT AE006471. The antibiotic resistance cassette is shown as an insert in gene PSLT044 and another IS*26* is shown inserted before gene PSLT064. The deleted sequence between PSLT064 and the point within *traD* gene is shown. The STTR10pl VNTR located intergenic between PSLT064 and PSLT065 (*ssbB*) is consequently deleted as well. Figure drawn using GenomeVx http://wolfe.ucd.ie/GenomeVx/ [17].

The isolate missing STTR10pl but with the AMP STR SUL resistance profile (09ST03520, MLVA 4-14-11-0-0211, S2 Table) had contigs identical to contigs C and D and the IS*26* contig in 10ST02333. The contig which corresponded most closely with contig B in 10ST02333 started at exactly the same point in the PSLT104 gene but finished a little shorter than in 10ST02333 at nt 36705–36712 in LT2 pSLT which is within the PSLT044 gene. The other contig was much shorter than contig A in 10ST02333 starting in PSLT044 and finishing just before PSLT064 at nt 53350 in LT2 pSLT. Consequently the insertion of the AMP STR SUL resistance cassette or of the IS*26* at PSLT064 (which may have been simultaneous events) has caused deletion of all of the genes from PSLT064 to the point within PSLT104. This includes many of the conjugative transfer genes. The theoretical construct for the 71167 bp pSLT of 09ST03520 was determined as detailed in S1 Text and as represented in Fig 3.

The theoretical locations for the single IS*26* elements in Fig 2 and Fig 3 have been confirmed by PCR using primers (S4 Table) which were located either side of the IS*26* element inserted into the *traD* gene, PSLT104, in pSLT Fig 2 and before the deleted sequence in pSLT Fig 3. The PCR products obtained would include the IS*26* sequence and were in the expected size range for the theoretical products.

### pSLT plasmids in fusion with other plasmids

We have found three isolates with plasmids which show evidence of fusion between pSLT plasmid sequence and sequence from another plasmid. One of these was a 145369 bp plasmid consisting of an intact IncX1 plasmid inserted into the *parB* gene (PSLT053 of LT2 pSLT) of an intact pSLT plasmid. It was derived after PacBio sequencing of a local strain of *S*. Typhimurium phage type U307 (01ST04081) isolated from a human in 2001. It belonged to the RG13 genotype [11]. The IncX1 component was one SNP different from the chromosomal IncX1 sequence in *S*. Berta SA20103550 (GenBank Acc. No. LHSR01000003) for two sequences, nt 4357–30052 and nt 31212–44417 in *S*. Berta. The intervening sequence nt 30053–31211 in *S*. Berta is replaced in the pSLT/IncX1 recombinant by a 12599 bp suite of genes including *tetA* and *tetR* genes for resistance to tetracycline and *dfrA* for resistance to trimethoprim. The pSLT component was one SNP different from a pSLT sequence in another RG13 genotype strain.

The other two fusion plasmids were two different examples of pSLT which have acquired significant lengths of IncI1 plasmid sequence. One was derived from isolate 15M663 (S2 Table) a sequenced strain of *S*. Typhimurium belonging to RG2 genotype isolated from a Danish patient in 2015 (Susanne Schjoerring, Statens Serum Institut, Denmark, personal communication). It was found to have very high sequence identity and coverage with pSTU288-1 (NC_021155), one of the plasmids found in *S*. Typhimurium str. U288 isolated from a pig in the UK and described as a hybrid plasmid with pSLT genes coupled with horizontally-acquired elements and containing gene cassettes conferring resistance against a number of antibiotics and compounds [18]. This strain also belonged to RG2 and had an MLVA profile almost the same as the Danish strain we have sequenced. Closer investigation showed that both strains had most of the pSLT sequence of LT2 but were missing nt 30107–36452 corresponding to genes PSLT040 to 045, including two of the virulence genes, *spvA* and *spvR*, and the sequence nt 19562–30010 was inverted (relative to the rest of the pSLT sequence) adjacent to the larger of the resistance gene cassettes. On the other side of the inverted sequence was an IS*26* insertion sequence linked to a *bla-*TEM-1 gene followed by a sequence which had 99% identity to most of the sequence nt 43611–75691 in the IncI1 plasmid R64 (AP005147) and was inserted into the pSLT sequence between PSLT026 and 027.

The other pSLT/IncI1 hybrid plasmid was found in a strain, L825 (DT193, S1 Table), included in the comparative genomics of *S*. Typhimurium study of Fu et al, 2017 [12]. This strain was isolated from a human in the UK in 2002 and was shown to belong to the RG9B genotype. The plasmid had most of the LT2 pSLT plasmid but was missing LT2 pSLT genes PSLT020 to 026. Inserted just after PSLT019 was a sequence with 99% identity to the sequence nt 29968–43819 in the IncI1 plasmid R64 barely overlapping with the R64 sequence in pSTU288-1 above. The insertion of the IncI1 genes therefore seems to have caused the deletion of the genes PSLT020 to 026. The L825 plasmid was almost identical to plasmids from six isolates of Typhimurium str. DT104 from Scotland isolated between 1990 and 1995 (Acc. Nos. CTCB01000014, CTBR01000013, CTLX01000014, CTMV01000030, CTOG01000013 and CTBP01000000), except that L825 lacked the gene cassette with tetracycline resistance genes. Examination of the CRISPR profiles of these isolates showed that although they were somewhat misassembled and not always complete they were much closer to the CRISPR profiles for RG9B genotypes than to RG8 genotypes to which DT104 isolates usually belong. Furthermore, the phage profiles of the DT104 isolates were the same as for L825 and another RG9B isolate L1849 [12] except that L825 lacked Gifsy-1. All these fusion plasmids showed that the pSLT component was closest in sequence to pSLTs belonging to hosts of the same genotype inferring that the plasmid fusion event has occurred inside the host carrying the pSLT.

## Conclusions

This study demonstrates the value of applying the data derived from whole genome sequencing of bacterial isolates towards the understanding of various aspects of plasmid evolution. Genetic characterisation of variants of the pSLT plasmid has provided evidence for co-evolution of pSLT plasmids and their *S*. Typhimurium hosts for both Australian and international isolates. This supports the proposition that the pSLT plasmid is most likely confined to its host cell since we find no examples of the same pSLT genotype occurring in different host genotypes. As a consequence the pSLT sequences which constitute a pSLT genotype are derived from hosts which all belong to one *S*. Typhimurium genotype. The study has also provided examples of pSLT plasmids showing large-scale changes under the influence of transposons or IS-elements as well as hybridisation with other plasmids to form fusion plasmids. The evidence indicates that these changes occur within the host cell of the pSLT and are maintained thereafter by vertical transmission.

## Materials and methods

### Bacterial strains and sequencing methods

Raw sequence files for 43 international and Australian strains of *Salmonella enterica* serovar Typhimurium with pSLT plasmids recruited for the comparative genomics study of Fu et al. 2017 [12] were downloaded from the European Nucleotide Archive website (https://www.ebi.ac.uk/ena) as in S1 Table. The genomes and pSLT sequences for seven reference strains were obtained from GenBank. Relevant genomic data and GenBank Accession numbers for the reference strains are shown in S1 Table. The GenBank numbers for the pSLT for the reference strains are shown in S3 Table. Another 21 international and Australian strains with pSLT plasmids and known genotype were chosen for the study (S2 Table). DNA was extracted from isolates grown overnight at 37°C on horse blood agar, using the QiaSymphony DSP DNA Mini kit (Qiagen) according to the manufacturer’s protocol. DNA was prepared for sequencing using the Nextera XT kit (Illumina) and sequenced on the NextSeq500 using the NextSeq 500 Mid Output v2 kit (300 cycles) (Illumina) according to the manufacturer’s instructions. Raw sequence files and associated metadata have been submitted to the European Nucleotide Archive with project accession number PRJEB25063.

For investigation of the 9 bp VNTR in the *traD* gene *S*. Typhimurium isolates with MLVA profiles representing most of the targeted genotypes were selected from our collection of locally isolated *Salmonella* strains. DT141 and DT141 var 1 isolates were also from this collection. They were presumed to belong to RG2 because all previously genotyped DT141 isolates belonged to RG2 and the MLVA profiles were consistent with those of the genotyped isolates.

### PCR procedures

Genotyping of the 21 additional sequenced strains was performed as per Hiley et al 2014 [11]. PCR amplification of the STTR10pl VNTR located in pSLT sequences was performed as per Hiley et al 2014 [11]. For PCR amplification of the 9 bp VNTR site in the *traD* gene the primers CAGTTCCTGTCCGACACCGCCT and GGATACAGAAGCGGTTGCGCCG were used (S4 Table). The mastermix contained 2 mM MgCl_2_, 5 pmol of each primer (Geneworks, Adelaide, South Australia) and 0.5 U of AmpliTaq Gold (Applied Biosystems, Foster City, Calif.); the initial cycling step was 94°C for 10 min followed by 30 cycles of 94°C for 30 s, 58.5°C for 45 s and 72°C for 1 m 30 s with a final 72°C for 10 min; a 6 μl aliquot from each PCR tube was electrophoresed in a 1.5% agarose gel containing 0.5 μg/ml ethidium bromide at 120 V for 40 min. Fragment sizing of PCR products was performed on an Applied Biosystems 3130 sequencer. PCR tests for the various pSLT genes and for confirming the proposed structure for plasmids in Fig 2 and Fig 3 were conducted using the primers listed (S4 Table) and the same procedure as for amplification of the 9 bp VNTR site and detection of PCR products but without application of a sequencer.

### Bioinformatic analysis

Sequences generated were quality trimmed using Trimmomatic v0.36 [19]. Chromosomal core SNPs were determined by mapping reads to the *S*. Typhimurium LT2 chromosome (NC_003197.2) using the Snippy v4.2 pipeline (https://github.com/tseemann/snippy), with the default parameters. Core SNPs were aligned and used to generate a maximum likelihood tree using the RAxML wrapper in Geneious R10 (Biomatters, New Zealand), using the GTR CAT model and 100 bootstrap replicates [20]. Snippy 4.2 was used to identify SNPs in the plasmid sequences using the *S*. Typhimurium LT2 pSLT plasmid (AE006471) as a reference, and running snippy-core to generate a full length alignment which was used to generate a maximum likelihood tree using the RAxML wrapper in Geneious R10 (Biomatters, New Zealand), using the GTR CAT model and 100 bootstrap replicates [20]. Sequences were *de novo* assembled into contigs using the SPAdes v3.10.1 assembler [21].

### PacBio sequencing and assembly methods

For strain 01ST04081 genomic DNA was extracted from a single colony grown overnight at 37°C on horse blood agar and sequenced on the PacBio RSII platform (Doherty Centre for Applied Microbial Genomics) using one SMRT cell, a 20 kb insert library and the P6 polymerase and C4 sequencing chemistry. *De novo* assembly of the raw PacBio sequencing data was done using the hierarchical genome assembly process (HGAP version 2) and quiver from the SMRT Analysis software suite (version 2.3.0 – http://www.pacb.com/devnet/) with default parameters. Next the sequence was polished using Pilon version 1.22. In brief, Illumina sequencing reads data were aligned back to the sequence and a new consensus was called thereby resolving any remaining sequencing errors. The GenBank Accession number for the pSLT from 01ST04081 is CP029841.

### Phage typing

Isolates were sent to the Microbiological Diagnostics Unit, University of Melbourne, Australia for phage typing by the Anderson scheme and antibiotic sensitivity testing.

### Statistical analysis

A chi-squared test for a two-way contingency table was applied.

## Supporting information

S1 Table International and Australian isolates from the panel recruited for the comparative genomics of *S*. Typhimurium study of Fu et al 2017 [12]. (DOCX)

S2 Table Additional local and international sequenced isolates of *S*. Typhimurium. (DOCX)

S3 Table The pSLT for seven reference strains of *S*. Typhimurium. (DOCX)

S4 Table Primers used for PCR tests. (DOCX)

S1 Text Determination of the theoretical constructs for pSLT sequences of *S*. Typhimurium isolates 10ST02333 and 09ST03520. (DOCX)
