# Supplementary material for: Genetic characterisation of variants of the virulence plasmid, pSLT, in Salmonella enterica serovar Typhimurium provides evidence of a variety of evolutionary directions consistent with vertical rather than horizontal transmission
Source: PLoS One. 2019 Apr 11;14(4):e0215207. doi: 10.1371/journal.pone.0215207 (PMC6459517; doi:10.1371/journal.pone.0215207)
Supplement: S1 Table — (DOCX) [file pone.0215207.s001.docx]

**S1 Table. International and Australian isolates from the panel recruited for the comparative genomics of *S.* Typhimurium study of Fu et al 2017 [12]**

| **Strain Name** | **Origin/Year** | **MLVA Euro** | **Phage Type/Genotype** | **Source** |
| --- | --- | --- | --- | --- |
| L818 | UK/Unknown | 2-14-18-9-0212 | 126/RG11 | GenBank BioProject No. PRJNA355598 |
| L825 | UK/Unknown | 5-10-0-12-0211 | Unknown/RG9B | As above |
| L1848 | Australia/2001 | 2-11-12-9-0212 | 135/RG12B | As above |
| L1849 | Australia/2004 | 4-12-20-7-0211 | 12a/RG9B | As above |
| L1852 | Australia/2006 | 2-10-10-9-0212 | 135a/RG12D/10A | As above |
| L1853 | Australia/2006 | 4-22-12-8-0211 | 104L/RG4B | As above |
| L1854 | Australia/2007 | 2-11-10-9-0212 | 135/RG12C | As above |
| L1855 | Australia/2007 | 3-13-8-8-0211 | 197/RG9A | As above |
| L1856 | Australia/2007 | 2-10-10-9-0212 | 135a/RG13 | As above |
| L1857 | Australia/2007 | 2-23-12-10-0212 | 9/RG14 | As above |
| L1858 | Australia/2007 | 4-14-14-11-0211 | 193/RG2 | As above |
| L1859 | Australia/2007 | 2-26-15-11-0212 | 8/RG14 | As above |
| L1860 | Australia/2007 | 3-12-14-14-0311 | 12/RG8 | As above |
| L1861 | Australia/2007 | 2-9-9-7-0212 | U302/RG10B | As above |
| L1863 | Australia/2007 | 3-17-10-15-0210 | 179/RG4A | As above |
| L1864 | Australia/2008 | 2-11-11-8-0212 | 102/RG12B | As above |
| L1865 | Australia/2008 | 2-10-11-9-0212 | 135a/RG13 | As above |
| L1867 | Australia/2008 | 2-9-9-5-0212 | 29/RG10B | As above |
| L1868 | Australia/2008 | 2-8-7-8-0212 | 186/RG10B | As above |
| L1869 | Australia/2008 | 2-8-7-8-0212 | 44/RG10A | As above |
| L1871 | Australia/2008 | 2-15-15-12-0212 | 126/RG11 | As above |
| L1872 | Australia/2008 | 2-13-15-8-0212 | 135/RG12D | As above |
| L1873 | Australia/2008 | 2-20-9-7-0212 | 135/RG12A | As above |
| L1877 | Australia/2009 | 2-11-10-9-0212 | 3/RG12C | As above |
| L1883 | Australia/2011 | 2-7-7-11-0212 | 170/RG13 | As above |
| SARA1 | Mexico | 4-12-8-10-0211 | Unknown/RG2 | SAMN03470046 |
| SARA4 | USA | 2-9-11-5-0212 | 20/RG12D/10A | SAMN03470049 |
| SARA5 | Mongolia | 4-14-8-7-0211 | Unknown/RG2 | SAMN03470050 |
| SARA6 | USA | 2-19-9-12-0212 | Unknown/RG12A | SAMN03470051 |
| SARA10 | USA | 14-13-NA-7-0111 | Unknown/ Not assigned | SAMN03470055 |
| SARA11 | Thailand | 3-13-18-26-0311 | Unknown/RG8 | SAMN03470056 |
| SARA12 | USA | 2-5-4-9-0109 | Unknown/RG11A/15 | SAMN03470057 |
| SARA15 | USA | 2-26-8-9-0212 | Unknown/RG12A | SAMN03470060 |
| SARA19 | Mexico | 4-13-10-7-0211 | Unknown/RG2 | SAMN03470064 |
| SARA20 | France | 4-12-11-7-0211 | Unknown/RG2 | SAMN03470065 |
| SARA21 | USA | 6-17-21-18-0011 | Unknown/ Not assigned | SAMN03470066 |
| 14028S | USA | 2-21-9-7-0212 | Unknown/RG12A | NC_016856 |
| D23580 | Malawi | 2-7-7-9-0210 | Untypable/RG12B | FN44405 |
| DT2 | UK | 2-17-6-9-0212 | 2/Not assigned | NC_022544 |
| LT2 | USA | 4-13-13-10-0211 | 4/RG2 | NC_003197 |
| SL1344 | USA | 2-8-8-7-0212 | 44/RG10A | NC_016810 |
| UK-1 | USA | 2-26-8-12-0212 | 1/RG12A | NC_016863 |
| VNP20009 | USA | 2-20-9-7-0212 | Unknown/RG12A | CP007804 |
| DT2B | UK | Not done | 2/RG12B | ERS007580 |
| DT8 | UK | Not done | 8/RG14 | ERS007592 |
| DT24 | UK | Not done | 24/RG12B | ERS007582 |
| DT99 | UK | Not done | 99/RG11A/15 | ERS007596 |
| DT135 | UK | Not done | 135/RG12D | ERS007567 |
| DT195 | UK | Not done | 185/RG2 | ERS007578 |
| U302 | UK | Not done | U302/RG8 | ERS007606 |
