# Supplementary material for: Genetic characterisation of variants of the virulence plasmid, pSLT, in Salmonella enterica serovar Typhimurium provides evidence of a variety of evolutionary directions consistent with vertical rather than horizontal transmission
Source: PLoS One. 2019 Apr 11;14(4):e0215207. doi: 10.1371/journal.pone.0215207 (PMC6459517; doi:10.1371/journal.pone.0215207)
Supplement: S2 Table — (DOCX) [file pone.0215207.s002.docx]

**S2 Table. Additional local and international sequenced isolates of *S*. Typhimurium**

| **Strain Name** | **Origin/Year** | **MLVA Euro** | **Phage Type/Genotype** | **ENA Accession** |
| --- | --- | --- | --- | --- |
| 01ST04081 | Australia/2001 | 2-10-9-11-0212 | U307/RG13 | ERS2213007 |
| 08ST00576 | Australia/2008 | 2-10-11-10-0212 | 6 var 1/RG13 | ERS2213008 |
| 08ST06126 | Australia/2008 | 4-14-8-8-0211 | 6 var 1/RG9 | ERS2213009 |
| 09ST00748 | Australia/2009 | 2-11-10-9-0212 | 6 var 1/RG13 | ERS2213010 |
| 09ST01531 | Australia/2009 | 2-10-9-12-0212 | 6 var 1/RG13 | ERS2213011 |
| 09ST01733 | Australia/2009 | 4-13-NA-10-0211 | 6 var 1/RG9 | ERS2213012 |
| 09ST03440 | Australia/2009 | 4-14-11-9-0211 | 141/RG2 | ERS2213013 |
| 09ST03520 | Australia/2009 | 4-14-11-0-0211 | 141/RG2 | ERS3215320 |
| 10ST00233 | Australia/2010 | 4-12-14-11-0211 | 6/RG2 | ERS2213014 |
| 10ST01093 | Australia/2010 | 4-10-10-8-0211 | 6 var 1/RG9 | ERS2213015 |
| 10ST02333 | Australia/2010 | 5-14-9-10-0211 | 141/RG2 | ERS2213016 |
| 10ST03749 | Australia/2010 | 2-10-12-10-0212 | 6/RG13 | ERS2213017 |
| 10ST07093 | Australia/2010 | 2-11-10-11-0212 | U307/RG13 | ERS2213018 |
| 11ST03440 | Australai2011 | 2-10-11-8-0212 | 6/RG13 | ERS2213019 |
| 11ST04232 | Australia/2011 | 2-10-11-8-0212 | 6/RG13 | ERS2213020 |
| P212_15 | Australia/2015 | 2-10-10-11-0212 | U307/RG13 | ERS2213021 |
| 15M662 | International/2015 | 2-15-7-9-0212 | Unknown/RG12D | ERS3215315 |
| 15M663 | Denmark/2015 | 5-19-9-11-0211 | Unknown/RG2 | ERS3215316 |
| 15M665 | Denmark/2015 | 3-18-14-15-0311 | Unknown/RG8 | ERS3215317 |
| 15M667 | Denmark/2009 | 3-16-15-24-0311 | Unknown/RG8 | ERS3215318 |
| 15M668 | Denmark/2015 | 4-17-13-9-0111 | Unknown/RG2 | ERS3215319 |
