# Supplementary material for: Genetic characterisation of variants of the virulence plasmid, pSLT, in Salmonella enterica serovar Typhimurium provides evidence of a variety of evolutionary directions consistent with vertical rather than horizontal transmission
Source: PLoS One. 2019 Apr 11;14(4):e0215207. doi: 10.1371/journal.pone.0215207 (PMC6459517; doi:10.1371/journal.pone.0215207)
Supplement: S3 Table — (DOCX) [file pone.0215207.s003.docx]

S3 Table. The pSLT for seven reference strains of *S.* Typhimurium

| Reference Strain | pSLT GenBank Accession Number |
| --- | --- |
| SL1344 | NC_017720 |
| D23580 | NC_013437 |
| 14028S | CP001362 |
| VNP20009 | CP008745 |
| UK-1 | CP002615 |
| DT2 | NZ_LN999012 |
| LT2 | AE006471 |
