# Supplementary material for: Genetic characterisation of variants of the virulence plasmid, pSLT, in Salmonella enterica serovar Typhimurium provides evidence of a variety of evolutionary directions consistent with vertical rather than horizontal transmission
Source: PLoS One. 2019 Apr 11;14(4):e0215207. doi: 10.1371/journal.pone.0215207 (PMC6459517; doi:10.1371/journal.pone.0215207)
Supplement: S4 Table — (DOCX) [file pone.0215207.s004.docx]

**S4 Table. Primer sequences used for detection by PCR of pSLT 9 bp VNTR and various pSLT genes**

| **Primer Name** | **Sequence (5 ’- 3’)** | **Target** | **Source** |
| --- | --- | --- | --- |
| DT104 Pl 9 bp VNTR-F | CAGTTCCTGTCCGACACCGCCT | 9 bp VNTR *traD* | NC_022570 |
| DT104 Pl 9 bp VNTR-R | GGATACAGAAGCGGTTGCGCCG |  |  |
| PSLT0111-F | GGAAGGTGAAGAAACAGCAG | PSLT0111-PSLT001 | AE006471 |
| PSLT001-R | CCATCCAGTATGCTTTTCGTG |  |  |
| PSLT010-F | CCGGTCATCTGAGGTTATCC | PSLT010 | AE006471 |
| PSLT010-R | GAAATTACCGGGATACACGGC |  |  |
| PSLT023-F | CCGGCGGTAGTGAATACAGA | PSLT023 | AE006471 |
| PSLT023-R | AACTGACGCGTCTGTCTCTC |  |  |
| PSLT042-F | GGCTTATCGCCATGCTGATG | PSLT042 | AE006471 |
| PSLT042-R | CACTGACGTAGTGAACCCG |  |  |
| PSLT052-F | GGTGCTTCCATCATAGGTTG | PSLT052 | AE006471 |
| PSLT052-R | GAAGTACGCGATCACGATTC |  |  |
| PSLT064-F | GTCCGGACTGAATCTGGCGG | PSLT064 | AE006471 |
| PSLT064-R | CAGCGACAGGCAGAATGCATC |  |  |
| PSLT065-F | GCAACGTCAACGGCAGGCTTG | PSLT065 | AE006471 |
| PSLT065-R | CTTGGACAGCCAGTGCGGC |  |  |
| PSLT073-F | GTGGAAAAACGACGCCAGGAA | PSLT073 | AE006471 |
| PSLT073-R | CATGAGGGCTGAGAGATCCT |  |  |
| PSLT091-F | GACATGCTGGCGGTGATTATGCA | PSLT091 | AE006471 |
| PSLT091-R | TCTGGCGTTTCATCCACGACAC |  |  |
| PSLT0103-F | CCAGTGACCGGACGGTGTATC | PSLT0103 | AE006471 |
| PSLT0103-R | CTTCCACCATCGCATCAGCG |  |  |
| PSLT0105-F | GCAAGCTCTGCCCTGATCTG | PSLT0105 | AE006471 |
| PSLT0105-R | ACGGGAACAACGTCAGCGC |  |  |
| PSLT0106-F | TCAGCTCCAGTCCTCAGTTC | PSLT0106 | AE006471 |
| PSLT0106-R | ACCTGAACCAGGGGAGAATG |  |  |
| ST03520 NODE 39-F | GGTTTCCATTTCTGTCGGAGGATG | Spans IS*26* contig A to contig B 09ST03520 | 09ST03520 |
| ST02333 NODE 27-R | GCTTTCTCGCCATAGATATCCTCC |  | 10ST02333 |
| ST02333 NODE 25-F | GGTTCTTCTGTGATGAGTTGCCCA | Spans IS*26* contig A to contig B 10ST02333 | 10ST02333 |
| ST02333 NODE 27-R | GCTTTCTCGCCATAGATATCCTCC |  | 10ST02333 |
