# Supplementary material for: Genetic characterisation of variants of the virulence plasmid, pSLT, in Salmonella enterica serovar Typhimurium provides evidence of a variety of evolutionary directions consistent with vertical rather than horizontal transmission
Source: PLoS One. 2019 Apr 11;14(4):e0215207. doi: 10.1371/journal.pone.0215207 (PMC6459517; doi:10.1371/journal.pone.0215207)
Supplement: S1 Text — (DOCX) [file pone.0215207.s005.docx]

**S1 Text. Determination of the likely constructs for pSLT sequences in 10ST02333 and 09ST03520**

*De novo* assembly of the sequences for the isolate with STTR10pl and the AMP STR SUL resistance profile (10ST02333) produced five contigs of interest (Plasmid 1 in Fig 2). One contig, A, spanned LT2 pSLT genes PSLT046 to within PSLT104 (*traD* ) at 100% identity. Another contig, B, spanned LT2 pSLT genes from within PSLT104 to PSLT045 with one SNP difference. Another contig, C, contained *sulII*, *strA* and *strB* resistance genes as part of a resistance cassette and another contig, D, contained a *bla*-TEM-1 gene. The fifth contig was the 820 bp IS element, IS*26* (Mollet et al). Blasting of the other four contigs with the IS*26* sequence showed that contigs B and D had 77 bp inverted repeats at the ends with the same sequence as the first 77 bp in the IS*26* contig while contigs A and C had 77 bp inverted repeats at the ends with the same sequence as the last 77 bp in the IS*26* contig. This was evidence that the IS*26* sequences are positioned between the four contigs A to D. Further evidence for this came from the eight bp target site duplications (TSDs) generated by the IS*26* insertion into the original pSLT genome (He et al). Thus the last eight bp at the right hand end of contig B before the first 77 bp of the IS*26* contig and the first eight bp at the left hand end of contig A after the last 77 bp of the IS*26* contig were the same as the sequence nt 37302 - 37309 in LT2 pSLT while the first eight bp at the left hand end of contig B after the first 77 bp (reversed) of IS*26* and the last eight bp at the right hand end of contig A before the last 77 bp (reversed) of IS*26 we*re the same sequence as nt 85689 - 85697 in LT2 pSLT. This has allowed us to construct the likely 103513 bp pSLT for the isolate (10ST02333) with both VNTR and the AMP STR SUL resistance profile **(Fig 2).** The inverted 77 bp repeats were removed from contig A to D during construction. It was reasoned that because the three antibiotic resistances appeared simultaneously in isolates that the two resistance cassettes most likely inserted as a single entity joined together by another IS*26* located at the insertion site nt 37302 - 37309 in LT2 pSLT and in order to join up the RH end of contig A with the LH end of contig B at nt 85689 - 85697 in the *traD* gene there would have to be another IS*26* at this point.

The isolate missing STTR10pl with the AMP STR SUL resistance profile (09ST03520) had contigs identical to contigs C and D and the IS*26* contig in 10ST02333. The contig which corresponded most closely to contig B in 10ST02333 started at exactly the same point in the *traD* gene but finished a little shorter than in 10ST02333 at nt 36705 - 36712 in LT2 pSLT which is within the PSLT044 gene and consequently it had a different TSD. The other contig was much shorter than contig A in 10ST02333 starting in PSLT044 and finishing just before PSLT064 at nt 53350 in LT2 pSLT. Consequently the insertion of the AMP STR SUL resistance cassette or of the IS*26* at PSLT064 (which may have been simultaneous events) has caused deletion of all of the genes from PSLT064 to the point within PSLT104. This includes many of the conjugative transfer genes. The pSLT construct for the isolate was estimated to be 71167 bp long and is shown **in Fig 3**.
